# Inpatient clinicians’ approach to diagnosis of urinary tract infections in older adults using the COM-B model: a qualitative assessment

**DOI:** 10.1017/ash.2024.401

**Published:** 2024-09-16

**Authors:** Sonali D. Advani, Nathan Boucher, Alison G.C. Smith, Connor Deri, Jillian E. Hayes, Rebekah Wrenn, Kenneth Schmader

**Affiliations:** 1 Division of Infectious Diseases, Department of Medicine, Duke University School of Medicine, Durham, NC, USA; 2 Sanford School of Public Policy, Duke University, Durham, NC, USA; 3 Department of Medicine, Duke University School of Medicine, Durham, NC, USA; 4 Department of Pharmacy, Duke University Hospital, Durham, NC, USA; 5 Duke Aging Center, Duke University School of Medicine, Durham, NC, USA; 6 Durham VA Medical Center, Durham, NC, USA

## Abstract

Our interviews of inpatient clinicians (physicians, physician assistants) modeled after the Capability, Opportunity, and Motivation Model of Behavior model revealed opportunity and motivation as important drivers for overdiagnosis and overprescribing for asymptomatic bacteriuria in older adults. Understanding these barriers is an important step toward implementing age-friendly stewardship interventions.

## Background

Asymptomatic bacteriuria (ASB) or the presence of positive urine cultures without symptoms referrable to the urinary tract is a common phenomenon in older adults (up to 50% incidence of ASB).^1^ Older adults frequently present to acute care hospitals with atypical symptoms (eg, confusion, functional decline) secondary to a myriad of noninfectious and infectious causes.^2,3^ Current guidelines recommend against testing and treating for bacteriuria in older adults in the absence of localizing or systemic signs of infection.^1^ Despite this, clinicians often order urine cultures and treat ASB with antibiotics in this population.^4,5^

Prior assessments of antimicrobial stewardship practices for ASB have primarily focused on assessing clinician knowledge, with little investigation into social, environmental, and cultural factors that influence these practices in hospitalized older adults.^5–7^ In this study, we applied the Capability, Opportunity, and Motivation Model of Behavior (COM-B) model^8,9^ to (1) understand how clinicians diagnose urinary tract infections (UTIs) in older adults (65 years and older) in inpatient settings and (2) assess drivers for prescribing antibiotics for ASB in this population.

## Methods

### Design and setting

We conducted semi-structured interviews of frontline clinicians at Duke University Hospital between August 1 and December 31, 2023. This study was deemed a quality improvement project by the Duke University Institutional Review Board.

### Recruitment

Eligibility criteria included (1) practicing as a clinician (attending physician, resident, or physician assistant) at Duke University Hospital and (2) having treated bacteriuria in an older adult (65 years or older) in the inpatient setting in the past 30 days. Eligible participants were identified with the help of chief residents and approached via electronic mail to participate. We stopped enrolling participants once new themes stopped emerging.

### Interview design

**Figure 1.**
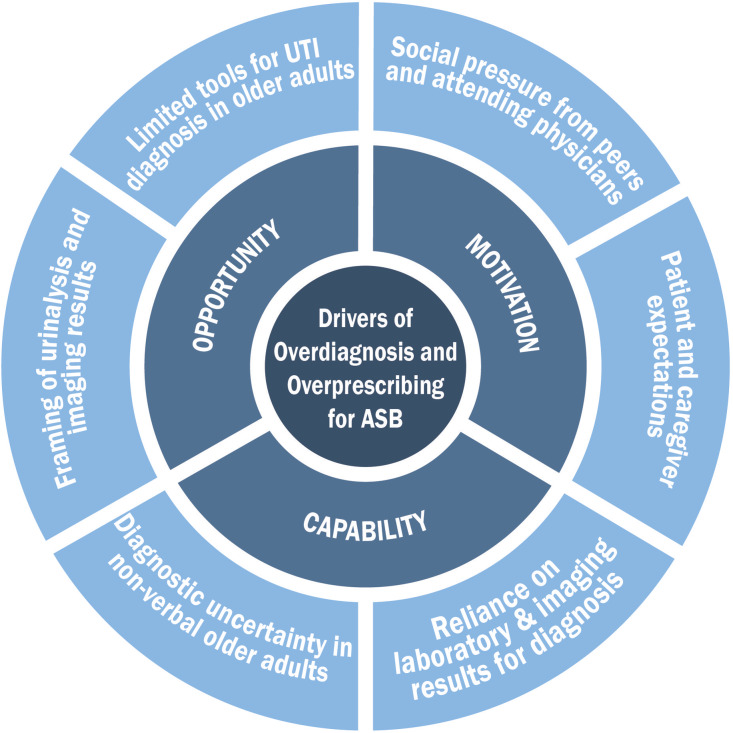
COM-B model of behavior change showing drivers of overdiagnosis and overprescribing for asymptomatic bacteriuria (ASB) in hospitalized older adults.

Our interview guide was created using the COM-B model^8,9^ of behavior change (Figure 1). This semi-structured interview guide included open-ended questions about the diagnosis and management of UTIs in hospitalized older adults and flexible probes based on participants’ responses (Supplement 1). The interview guide was piloted on 2 clinicians and revised based on feedback from the study team.

### Interview conduct

The principal investigator (SDA) trained in qualitative interviewing conducted all interviews. Participants were verbally consented prior to starting the interview. Interviews were conducted using Zoom videoconference technology, lasted 25–30 minutes, and were audio-recorded and transcribed with consent. Participants received a $25 gift card for completing the interview.

### Qualitative coding and analysis

A codebook was developed using the 6 COM-B components and their definitions (Figure 1).^9^ The codebook was piloted on the 2 interviews and subsequently updated with clarifications of how the COM-B components applied to specific questions. Transcripts were coded by one trained coder (SDA) using deductive coding (Supplement 2). Any questions about coding were discussed with the senior team member (NB). *Delve* qualitative data analysis software was used for coding and analysis.^10^

## Results

Fourteen of the 16 initially selected participants were interviewed (87.5% response rate). Table 1 displays the demographic characteristics of participants. Several themes emerged from the participant interviews, which we categorized based on the COM-B framework in Figure 1 and Supplement 3.

**Table 1.** Demographic characteristics of 14 participants

Characteristics	Number of participants, n (%)
**Age**	
30–40	12 (85.7)
40–50	2 (14.3)
**Female sex**	8 (57.1)
**Race**	
African American	1 (7.1)
Asian	3 (21.5)
White	9 (64.3)
Other	1 (7.1)
**Hispanic ethnicity**	3 (21.5)
**Role**	
Residents	10 (71.4)
Attending physician	2 (14.3)
Physician assistants	2 (14.3)
**Specialty**	
Internal medicine	9 (64.3)
Emergency medicine	3 (21.5)
Urology	2 (14.3)

### Theme 1 (Capability): Clinicians have the knowledge to appropriately diagnose a UTI

- You’re looking for specific symptoms: dysuria, suprapubic pain, nausea, vomiting, or abdominal pain.
- … can be lower or upper urinary symptoms, plus or minus the manifestation of systemic symptoms.
- First, I think about symptoms, so if they have symptoms consistent with a UTI, so like dysuria, increased frequency, or increased urgency …

### Theme 2 (Capability) Diagnostic uncertainty is a major driver of overprescribing in nonverbal hospitalized older adults

- They are elderly patients who are slightly altered, but we have a *CT*, and all they have is like a *bladder wall thickening*; everything else is negative. It’s possible that they still have a UTI.
- Patients who have so many other comorbidities, and if they’re elderly and sometimes they have communication challenges, it’s harder for us to get meaningful, reliable historical information.

### Theme 3 (Capability and Opportunity): Clinicians are influenced by positive urinalysis parameters and imaging findings to diagnose a UTI in nonverbal older adults

- Frank pyuria, …and it’s like nitrite positive like this, this is a UTI, right?
- If they have high whites in their urine, so like greater than 10.
- ..the main one that I look at is the white count, then it goes to the nitrite.
- Sometimes we’re seeing a CT result even before seeing the patient.

### Theme 4 (Motivation): Physicians-in-training are influenced by peers and attending physicians to treat ASB in hospitalized older adults

- My attendings… almost always err on the side of *starting with IV antibiotics,* just to be safe.
- There are some attendings who say, “Oh, that’s a high number in terms of white blood cells. Maybe you should probably just treat that despite them not being symptomatic.”
- Yeah, I think my colleagues, in general, are going to prescribe antibiotics almost every single time.

### Theme 5 (Motivation): Clinicians feel pressure from patients and caregivers to treat ASB in hospitalized older adults with antibiotics

- …definitely felt pressure from patients and their families.
- Many times, the family will say, Oh, I think he has a UTI because …when he was confused last time, the doctor found a UTI, gave them antibiotics, and they got better.

### Theme 6 (Opportunity): Clinicians believe a risk scoring system or calculator could help reduce inappropriate prescribing for bacteriuria in older adults

- If there is a scoring system that can help us to say low or high probability, I think that will be very useful.
- Yeah, I mean like a *validated scoring system* would be pretty awesome.
- If there was some sort of clinical support tool, whether it’s embedded in Epic, or it’s like an *MDCalc sort* of thing.

## Discussion

Our semi-structured interviews revealed that clinicians had the knowledge (capability) to diagnose UTIs but encountered diagnostic uncertainty when assessing hospitalized older adults with dementia or delirium. We identified several drivers of overdiagnosis and overprescribing for ASB in this population: (1) cultural reliance on urinalysis parameters and imaging results for UTI diagnosis, (2) influenced by antibiotic prescribing behavior of their peers and supervising staff physicians, (3) patient and caregiver expectations, and (4) lack of tools to help with diagnosis of UTIs in older adults.

This study is an important first step in understanding how prescribing clinicians make antibiotic prescribing decisions for bacteriuria in hospitalized older adults. Our findings revealed that the diagnostic uncertainty in these cases leads to an overreliance on urinalysis and imaging findings, influence by peer practice, and susceptibility to patient and caregiver expectations.

Our study has some limitations. First, participants self-reported their behavior, so their responses may not reflect their actual practice. Second, our findings reflect 14 inpatient clinicians at 1 teaching hospital. However, previous research has found that 9–17 interviews are adequate to obtain saturation.^11^

Past stewardship interventions to reduce antimicrobial prescribing for ASB have largely focused on education and used a one-size-fits-all approach across all populations.^7^ However, this study’s findings suggest that we need a nuanced approach to antibiotic stewardship for ASB in older adults, especially those with dementia and delirium. To reduce inappropriate antimicrobial prescribing for ASB in this population, we need to develop UTI prediction tools or personalized risk estimates while engaging patients and caregivers in shared decision-making.^3,4^

## Supporting information

Advani et al. supplementary material 1 Advani et al. supplementary material

Advani et al. supplementary material 2 Advani et al. supplementary material

Advani et al. supplementary material 3 Advani et al. supplementary material

## Supplementary material

To view supplementary material for this article, please visit https://doi.org/10.1017/ash.2024.401.
