# Supplementary material for: Inpatient clinicians’ approach to diagnosis of urinary tract infections in older adults using the COM-B model: a qualitative assessment
Source: Antimicrob Steward Healthc Epidemiol. 2024 Sep 16;4(1):e134. doi: 10.1017/ash.2024.401 (PMC11406559; doi:10.1017/ash.2024.401)
Supplement: Advani et al. supplementary material 1 — Advani et al. supplementary material [file S2732494X24004017sup001.docx]

Supplement 1: ***Interview Guide for Semi Structured Interview***

Thanks for agreeing to participate in this interview. The goal of this study is to learn more about your experience diagnosing UTIs in older adults. All of your responses will remain confidential, names and identifiers will not be used, and findings will only be reported in an aggregate manner. Additionally, please do not discuss any patient identifiers or other protected health information during the interview. If you agree to participate in the interview, we will assume that you have given us permission to audio record and transcribe this interview. You may choose to stop the interview at any time, and there is no penalty to you for not completing the interview. If you complete the interview, we will offer you a $25 Starbucks gift card to thank you for your time. We will audio record this interview for transcription and save the audio recordings and transcriptions (without any of your identifiers) in a secure Duke folder that is password protected. Do I have permission to begin the interview and audio record?

Do you have any questions before we begin?

Begin the recording…..

**A- Let’s start by discussing a little bit about your role in the hospital**

1. Can you describe your role in the hospital?

**B – Let’s move on to your current approach**

1. How do you diagnose a UTI in an older patient (65years or older)?

- prompt- if they have dementia

- prompt if they are altered

1. What prompts you to order urinalysis or urine culture in an older adult? (prompt- is there a risk/testing threshold)
2. What pieces of information do you use to make a decision about treating an older adult with dementia or delirium for a UTI? prompt- symptoms, labs, etc?
   1. Which laboratory values impact your diagnosis? (Prompt serum and urine labs?)
   2. Which urinalysis parameters do you rely on?
3. What are the challenges you perceive with diagnosing UTI in this population?
4. Have you heard the term ‘asymptomatic bacteriuria”? What does it mean to you?
5. How do you approach a patient with ASB? (Prompt- What prompts you to treat positive urine cultures in these patients?)
6. How do your colleagues approach an older adult with positive urine cultures?
7. What resources or support have you used for assessment or management of older patients with positive urine cultures if they have dementia or AMS? (prompt – EMR/pharmacist/ID consults)

**C - Questions about existing stewardship interventions**

1. Has a pharmacist or stewardship team at this hospital approached you to stop antibiotics in an older adult with positive urine cultures
2. Can you walk me through the most recent experience
3. How did this interaction make you feel? (Prompt – did it feel helpful, interruptive, etc)
4. How did your team approach this recommendation?
5. How did your patients or their family respond to the stewardship intervention emotionally? (Prompt-How did the intervention affect relationships with your patients?)

**D- Closing**

- What can we do to support you in management of older adults with dementia or AMS who present with positive urine cultures?
- Is there anything else you like to share or discuss related to this topic?

Stop the Audio recording

Thank interviewee for his/her time and for participating in the study. Please let me know we will mail out their gift card to them and that we just have some basic demographic questions that we need to ask now…

1. What is your gender?
   1. Man
   2. Woman
   3. Other (type in)
2. What is your age?
3. <30 years
4. 30-40 years
5. 41-50 years
6. 51-64 years
7. >=65 years
8. What is your ethnicity?
   1. Hispanic or Latino
   2. Non-Hispanic or Latino
9. What is your race? (Select all that apply)
   1. White
   2. Black or African America
   3. Asian
   4. American Indian or Alaska Native
   5. Native Hawaiians or other pacific islands
   6. Middle Eastern American
   7. Other (type in)
10. What is your specialty?
11. What is your level of training?
    1. Medical student
    2. Intern
    3. Resident
    4. Fellow
    5. Attending Physician (Not in Training)
    6. Advanced Practice Provider or Nurse practitioner (in training)
    7. Advanced Practice Provider or Nurse practitioner (not in training)
12. How many years have you worked at Duke (include years in training)
