# Supplementary material for: Inpatient clinicians’ approach to diagnosis of urinary tract infections in older adults using the COM-B model: a qualitative assessment
Source: Antimicrob Steward Healthc Epidemiol. 2024 Sep 16;4(1):e134. doi: 10.1017/ash.2024.401 (PMC11406559; doi:10.1017/ash.2024.401)
Supplement: Advani et al. supplementary material 2 — Advani et al. supplementary material [file S2732494X24004017sup002.docx]

| **Supplement 2: Codebook** | | | | |
| --- | --- | --- | --- | --- |
| **Theme** | **Subtheme** | **Code** | **Subcode** | **When to Use** |
| Capability | Psychological Capability | UTI Diagnosis | Older adult | When interviewee describes how they diagnose UTI |
|  |  |  | Dementia or Delirium |  |
|  |  |  |  |  |
| Capability | Psychological Capability | Urine Test ordering | Older adult | When the interviewee describes situations in which they order urine tests |
|  |  |  | Dementia or Delirium |  |
|  |  |  | Urinalysis |  |
|  |  |  | Urine culture |  |
|  |  |  |  |  |
| Capability | Psychological Capability | Treatment Decisions | Symptoms | When the interview describes signs or symptoms that influence treatment |
|  |  |  | Urine Test Results | When the interview describes urine test results that influence treatment |
|  |  |  | Imaging | When the interview describes imaging results that influence treatment |
|  |  |  |  |  |
| Capability | Psychological Capability | Urinalysis Interpretation | Pyuria | When the interviewee describes how they interpret urine tests |
|  |  |  | Nitrite |  |
|  |  |  | Squams |  |
|  |  |  | Other UA parameters |  |
|  |  |  |  |  |
| Opportunity | Social Opportunity | Challenges in diagnosis | Patient presentation related | When the interviewee describes specific aspects of patient presentation that pose a challenge to diagnosing UTI |
|  | Physical Opportunity |  | Health system related | When the interviewee describes specific setting related challenges (e.g. access to tools, etc) that pose a challenge to diagnosing UTI |
|  |  |  | Nursing | When interviewee describes specific challenges with obtaining cultures related to nursing |
|  |  |  | Time | When interviewee describes lack of time |
|  |  |  | Laboratory | When interviewee describes how framing of test results influences prescribing |
|  |  |  |  |  |
| Capability | Psychological Capability | ASB understanding | Patient type | When the interviewee describes what patients have ASB |
|  |  |  | Incidence |  |
|  |  |  |  |  |
| Motivation | Reflexive Motivation | Approach to ASB |  | When interviewee describes whether they treat or don't treat ASB patients with antibiotics |
|  |  |  |  |  |
| Opportunity | Physical Opportunity | Stewardship interaction |  | When interviewee describes the stewardship interaction |
|  |  |  |  |  |
| Motivation | Automatic Motivation | Stakeholder expectations | Patient | When interviewee describes patient expectations around treatment or response to stopping treatment. |
|  |  |  | Family | or family |
|  |  |  | Attending | or attending and peers |
|  |  |  | Nurse | or nurse |
|  |  |  |  |  |
| Opportunity | Physical Opportunity | Tool | Current tools | When interviewee described which tools and resources they use for diagnosis and management of UTIs |
|  |  |  | Future tools | When interviewee describes which tools and resources they would like to use for diagnosis and management of UTIs |
